# Identification of *mcr-8* in Clinical Isolates From Qatar and Evaluation of Their Antimicrobial Profiles

**DOI:** 10.3389/fmicb.2020.01954

**Published:** 2020-08-24

**Authors:** Nahla O. Eltai, Brianna Kelly, Hassan A. Al-Mana, Emad B. Ibrahim, Hadi M. Yassine, Asmaa Al Thani, Muna Al Maslmani, Christine Lammens, Basil B. Xavier, Surbhi Malhotra-Kumar

**Affiliations:** ^1^Biomedical Research Centre, Qatar University, Doha, Qatar; ^2^Laboratory of Medical Microbiology, Vaccine and Infectious Disease Institute, University of Antwerp, Antwerp, Belgium; ^3^Laboratory Medicine and Pathology, Hamad General Hospital, Doha, Qatar; ^4^Infectious Disease Department, Hamad General Hospital, Doha, Qatar

**Keywords:** *mcr-8.1*, *mcr-1.1*, colistin, Qatar, *K. pneumoniae*, *E. coli*, *P. aeruginosa*

## Abstract

This study was performed to investigate the genotypic causes of colistin resistance in 18 colistin-resistant *Klebsiella pneumoniae* (*n* = 13), *Escherichia coli* (*n* = 3) and *Pseudomonas aeruginosa* (*n* = 2) isolates from patients at the Hamad General Hospital, Qatar. MIC testing for colistin was performed using Phoenix (BD Biosciences, Heidelberg, Germany) and then verified with SensiTest Colistin (Liofilchem, Zona Ind. le, Italy). Strains determined to be resistant (MIC > 4-16 μg/mL) were then whole-genome sequenced (MiSeq, Illumina, Inc.). Sequences were processed and analysed using BacPipe v1.2.6, a bacterial whole genome sequencing analysis pipeline. Known chromosomal modifications were determined using CLC Genomics Workbench v.9.5.3 (CLCbio, Denmark). Two *K. pneumoniae* isolates (KPN-15 and KPN-19) harboured *mcr-8.1* on the IncFII(K) plasmids, pqKPN-15 and pqKPN-19, and belonged to ST383 and ST716, respectively. One *E. coli* isolate harboured *mcr-1.1* on the *IncI2* plasmid pEC-12. The other 15 isolates harboured known chromosomal mutations linked to colistin resistance in the PhoPQ two-component system. Also, three *K. pneumoniae* strains (KPN-9, KPN-10 and KPN-15) showed disruptions due to IS elements in *mgrB*. To our knowledge, this marks the first description of *mcr-8.1* in *K. pneumoniae* of human origin in Qatar. Currently, more research is necessary to trace the source of *mcr-8.1* and its variants in humans in this region.

## Introduction

Resistance to colistin, a last-resort antibiotic, has been identified in humans, livestock animals, and the environment (Forde et al., 2018). Colistin (polymyxin E) is a cationic polypeptide synthesised from *Paenibacillus polymyxa* subspecies C*olistinus* (Poirel et al., 2017). Colistin was first introduced for the treatment of GNB (Gram-negative bacteria) infections in 1952 and was used until the 1980s; however, its use in human medicine was limited due to neuro and nephrotoxicity (Akajagbor et al., 2013). Unfortunately, the emergence of multi and extensive drug resistance pathogens has necessitated colistin’s reintroduction to clinical practice (Catchpole et al., 1997). Colistin resistance is typically caused by chromosomally mediated modifications to its target site, the LPS (lipopolysaccharide) in the outer membrane of GNB, thereby reducing its binding affinity (Poirel et al., 2015; Aghapour et al., 2019). Colistin resistance was thought to have only a vertical spread, but recently horizontally transmitted plasmid-mediated resistance has emerged.

Colistin is a cationic molecule that interactions with the negatively charged lipopolysaccharide present on the outer membrane. This increases membrane permeability, eventually leading to bacterial death (Poirel et al., 2015). Interestingly, colistin resistance has been linked to higher levels of susceptibility to other antimicrobial agents, due to the outer membrane disruption enabling better passage of these into the cell. This appears to be particularly true for hydrophobic antibiotics (Li et al., 2020). Conversely, colistin resistance has also been implicated in resistance to cationic antimicrobial peptides present in the host. Studies have documented that *mcr-1.1* can confer resistance to the cationic lysozyme as well as other cationic host antimicrobials (Napier et al., 2013; Sherman et al., 2016). This presents a significant concern due to the potential of *mcr* genes to be maintained due to the advantage they provide in surviving host-microbial (Napier et al., 2013; Sherman et al., 2016).

Transferable resistance was first reported in China in 2015 as the *mcr-1.1* gene, a phosphoethanolamine (pEtN) transferase which adds pEtN to the lipid A moiety of the LPS, preventing colistin binding through the reduction of the negative charge on the LPS (Liu et al., 2016; Aghapour et al., 2019). Homologous pEtN transferases were identified in Gram-negative bacteria, such as *mcr-2.1* from Belgian porcine *E. coli* (Xavier et al., 2016). Since then, nine different *mcr* genes and several variants have been identified across the globe (Carroll et al., 2019). In Qatar, *mcr-1.1* was first reported in 2018 in *E. coli* isolated from broiler chickens and MDR *E. coli* from a patient with subarachnoid haemorrhage (Eltai et al., 2017; Forde et al., 2018). Additionally, studies have described *mcr-1.1* from clinical isolates in Jordan and *mcr-5.1* present in human isolates in Saudi Arabia (Redhwan et al., 2019). *Mcr-8.1* has been previously reported in animal, and human *Klebsiella pneumoniae* isolates from China and Laos, and recently, Algeria, Morocco, France, Bangladesh, and Saudi Arabia (Wang et al., 2018, 2019; Hadjadj et al., 2019; Bonnin et al., 2020; Farzana et al., 2020; Hala et al., 2020; Nabti et al., 2020). A recent review on colistin resistance in the Middle East reported the presence of *mcr* genes in six countries in the region in *K. pneumoniae* isolates (Aris et al., 2020). Interestingly, it is reported that the majority of resistance is mediated by *mgrB* mutations with few appearing to harbour plasmid-mediated resistance. However, the proportion of resistance due to *mcr* gene has been increasing since their discovery in 2015. This increase in resistance has been suggested the use of antibiotics in the region (Aris et al., 2020).

While primarily present in animal associated isolates *mcr* genes are a typical example of the importance and necessity for a One Health approach, with recent reports showing carriage of *mcr* genes in both hospital and community-associated human bacterial pathogens such as *K. pneumoniae* and *Acinetobacter* spp. (Agaba et al., 2017). Therefore, surveillance of antimicrobial resistance mandates the determination of the prevalence of *mcr* in human bacterial pathogens and an assessment of their impact on clinical outcome. In this study, we analysed 18 isolates obtained from patients admitted to the Hamad General Hospital (HGH) in Qatar in order to determine the prevalence and identify the underlying mechanisms leading to their colistin resistance.

## Materials and Methods

In total, 18 isolates belonging to *K. pneumoniae* (*n* = 13), *E. coli* (*n* = 3) and *Pseudomonas aeruginosa* (*n* = 2) were analysed in this study. Strain characteristics of the 18 isolates are described in Table 1 and Supplementary Table S1. Species confirmation was performed by MALDI-TOF (Bruker Daltonik GmbH, Leipzig, Germany). Ethical approval (MRC-01-17-198) was obtained from the Medical Research Centre (MRC), Hamad Medical Corporation (HMC), Doha, Qatar. Initial antimicrobial susceptibility testing was performed by Phoenix using the NMIC/ID-5 panel (BD Biosciences, Heidelberg, Germany) according to the manufacturer’s recommendations. Briefly, panels were inoculated with 0.5 McFarland pure culture, placed into the instrument and incubated at 35°C. The instrument tests panel every 20 min up to 16 h if necessary. MIC values of each antimicrobial agent automatically read as susceptible, intermediate, or resistant (SIR). Isolates showing colistin resistance by Phoenix were consequently confirmed using the SensiTest colistin kit (Liofilchem, Zona Ind. le, Italy), following the manufacturer’s instructions. Briefly, a suspension of 0.5 McFarland standard was prepared from the tested organism, and then diluted 1:20 in saline. 0.4 ml of this diluted suspension was added to a tube of Muller Hinton broth provided in the kit (solution B). 100 μl of solution B was dispensed to the seven wells in a row. Then the plate was incubated at 36 ± 2°C for 16-20 h. The lowest concentration of antibiotic that inhibits visible growth was recorded and interpreted according to Clinical and Laboratory Standards Institute (CLSI) guidelines.

**TABLE 1:** Mutations present in isolates (*n* = 18) of *K. pneumoniae* (*n* = 13), *E. coli* (*n* = 3), and *P. aeruginosa* (*n* = 2) conferring resistance to colistin and other antimicrobials.

**Strain ID**	**Sample type**	**DOI**	**Organism**	**ST**	**Gene modifications leading to colistin resistance**		**Colistin MIC**				**Other resistance genes**
				
					**Chromosomal**	**Plasmid mediated**	**Phoenix**		**SensiTest**		
KPN-2	Wound	August, 2018	*K. pneumoniae*	ST792	PhoP L26Q	NA	>4	R	0.5	S	Beta-lactam: *blaSHV-26*, Fosfomycin: *fosA*, Quinolone: *oqxB, oqxA*
KPN-3	Urine	July, 2018	*K. pneumoniae*	ST25	PhoP L26Q	NA	>4	R	>16	R	Beta-lactam: *blaSHV-81*, Fosfomycin: *fosA*, Quinolone: *qnrS, oqxB, oqxA*, Macrolide: *mph(A)* Trimethoprim: *dfra15, dfrA1* Phenicol: *catA1* Aminoglycoside: *aph (6)-Id, aph(3′*)-*Ib*, Sulphonamide: *sul1, sul2*, Tetracycline: *tet(A), tet(D)*
KPN-4	Urine - Bladder	January, 2018	*K. pneumoniae*	ST323	PhoP L26Q	NA	>4	R	>16	R	Beta-lactam: *blaSHV-99*, Fosfomycin: *fosA*, Quinolone: *oqxA, oqxB*
KPN-5	Wound (post-operative)	September, 2018	*K. pneumoniae*	ST11	PhoP L26Q	NA	>4	R	>16	R	Beta-lactam: *blaSHV-182*, *blaCTX-M-15, blaOXA-232, blaTEM-1B*, Fosfomycin: *fosA*, Quinolone: *aac (6′)-Ib-cr, oqxA, oqxB* Aminoglycoside: *rmtF, aph (3′)-Ib, aac(6′)-Ib3, aph(6)-Id* Sulphonamide: *sul2*
KPN-9	Urine - Catheter	August, 2018	*K. pneumoniae*	ST2096	PhoP L26Q, mgrB interruption ISKpn26 (IS5) at 74th base	NA	>4	R	>16	R	Beta-lactam: *blaSHV-28, blaOXA-1, blaOXA-232*, Fosfomycin: *fosA*, Quinolone: *aac (6′)-Ib-cr, oqxA, oqxB*, Macrolide: *mph(E), msr(E)*, Trimethoprim: *dfrA12, dfrA1*, Phenicol: *catB3*, Aminoglycoside: *aadA2, armA*, Sulphonamide: *sul1*
KPN-10	Tracheal Aspirate	May, 2018	*K. pneumoniae*	ST2096	PhoP L26Q, mgrB frameshift due to insertion at 74th base	NA	>4	R	16	R	Beta-lactam: *blaTEM-1A, blaCTX-M015, blaSHV-28, blaOXA-1*, Fosfomycin: *fosA*, Quinolone: *oqzA, oqxB*, Macrolide: *mph(E), msr(E)*, Trimethoprim: *dfrA12, dfrA1, dfrA14* Phenicol: *catB3*, Aminoglycoside: *aac(6′)-IB-cr, aadA, armA* Sulphonamide: *sul1* Tetracycline: *tet(D)*
KPN-14	Tissue	November, 2016	*K. pneumoniae*	ST383	PhoP L26Q, mgrB L9*	NA	>4	R	>16	R	Beta-lactam: *bla)XA-48, blaCTX-M-14, blaSHV-26*, Fosfomycin: *fosA*, Quinolone: *oqxB, oqxA*, Macrolide: *mph(A), msr(E), erm(B)*, Trimethoprim: *dfrA6*, Phenicol: *catA1*, Aminoglycoside: *armA, aph(6)-Id, aph(3′′)-Ib, aph(3′)-Ia*
KPN-15	Urine - Catheter	October, 2017	*K. pneumoniae*	ST383	PhoP L26Q, *mgrB* interruption at 118th base	*mcr-8.1*	>4	R	>16	R	Beta-lactam: *blaSHV-26*, Fosfomycin: *fosA*, Quinolone: *oqxA, oqxB*, Trimethoprim: *dfrA12, dfrA1*, Phenicol: *catA1*, Aminoglycoside: *aadA2*, Sulphonamide: *sul1*
KPN-16	Urine - Catheter	June, 2017	*K. pneumoniae*	ST383	PhoP L26Q	NA	>4	R	8	R	Beta-lactam: *blaCTX-M-15, blaSHV-26, blaOXA-9, blaCTX-M-14b, blaTEM-1B, blaNDM-5, blaOXA-48*, Fosfomycin: *fosA*, Quinolone: *qnrS1, oqxB, oqxA, mph (E), aac (6′)-Ib-cr, msr(E), mph(A)*, Trimethoprim: *dfrA5*, Phenicol: *catA1*, Aminoglycoside: *aadA1, aph(3′)-Ia, aac(6′′)-Ib, aph(6)-Id, aph(3′)-Ib, aph(3′)-VI, armA* Sulphonamide: *sul1, sul2*, Tetracycline: *tet(A)*
KPN-17	ETA	August, 2017	*K. pneumoniae*	ST231	PhoP L26Q	NA	>4	R	16	R	Beta-lactam: *blaTEM-Ib, blaCTX-M-015, blaOXA-232*, Fosfomycin: *fosA*, Quinolone: *oqxB, oqxA*, Macrolide: *mph(A), erm(B)*, Trimethoprim: *drfA12*, Phenicol: *catA1*, Aminoglycoside: *aac (6′)-Ib3*, *aadA2, aac (6′)-Ib-cr, qepA4, rmtF* Sulphonamide: *sul1*, Tetracycline: *tet(B)*, Rifampicin: *ARR-3*
KPN-18	Wound	October, 2015	*K. pneumoniae*	ST147	PhoP L26Q	NA	>4	R	16	R	Beta-lactam: *blaCTX-M-15, blaSHV-67*, Fosfomycin: *fosA*, Quinolone: *aac(6′)-Ib-cr, oqxA, oqxB, qnrB1*, Trimethoprim: *dfrA12*, Aminoglycoside: *rmtF, aac(6′)-Ib3, aadA2*, Sulphonamide: *sul1*
KPN-19	Bronchial Wash	December, 2015	*K. pneumoniae*	ST716	PhoP L26Q	*mcr-8.1*	>4	R	16	R	Beta-lactam: *blaTEM-Ib, blaCTX-M-15, blaSHV-27, blaOXA-1*, Fosfomycin: *fosA*, Quinolone: *oqxA, oqxB, qnrB1*, Macrolide: *mph(A)*, Trimethoprim: *dfrA12, dfrA14*, Phenicol: *catB3*, Aminoglycoside: *aac(3)-Iia, aac(6′)-Ib-cr, aph(3′)-Ib, aph(3′′)-Ia, aph(6)-Id, aadA2*, Sulphonamide: *sul1, sul2*, Tetracycline: *tet(A)*
KPN-20	Blood	February, 2016	*K. pneumoniae*	ST231	PhoP L26Q Absence of mgrB	NA	>4	R	< 16	R	Beta-lactam: *blaTEM-iB, blaCTX-M-15, blaSHV-28*, Fosfomycin: *fosA*, Quinolone: *oqxA, oqxB*, Macrolide: *mph(A), erm(B)* Trimethoprim: *dfrA12*, Phenicol: *catA1* Aminoglycoside: *aac(6′)-Ib3, aac (6′)-Ib-cr, aadA2*, Sulphonamide: *sul1*
EC-12	Wound – Drainage	May, 2016	*E. coli*	ST156	NA	*mcr-1.1*	4	R	4	R	Beta-lactam: *blaCTX-M-55, blaTEM-1B*, Fosfomycin: *fosA*, Quinolone: *qepA1*, Macrolide: *mph (E), mdf(A)*, Trimethoprim: *dfraA17*, Phenicol: *catA1*, Aminoglycoside: *aph (3′)-Ib, aadA5, aph(6)-Id*, Sulphonamide: *sul1, sul2* Tetracycline: *tet(A)*
EC-21	Urine	August, 2018	*E. coli*	ST1193	NA	NA	2	R	4	R	Beta-lactam: *blaCTX-M-15, blaOXA-1* Macrolide: *mdf(A)*, Phenicol: *catB3*, Aminoglycoside: *aac(3)-IIa, aac(6′)-Ib-cr*
EC-22	Urine	September, 2018	*E. coli*	ST452	NA	NA	4	R	4	R	Macrolide: *mdf(A)*
PA-11	Sputum	March, 2015	*P. aeruginosa*	ST500	NA	NA	4	I	2	S	Beta-lactam: *blaOXA-486, blaPAO*, Fosfomycin: *fosA*, Quinolone: *crpP*, Phenicol: *catB7*, Aminoglycoside: *aph(3′)-IIb*
PA-13	Bone tissue	October, 2019	*P. aeruginosa*	ST357	NA	NA	≤1	X	4	R	Beta-lactam: *blaVEB-1, blaOXA-10, blaPAO, blaNDM-1, blaOXA-50*, Fosfomycin: *fosA*, Quinolone: *crpP*, Trimethoprim: *dfrB2*, Phenicol: *catB7, cmlA1*, Aminoglycoside: *aph(3′)-Iib, aadA1, aph(3′)-VI, ant(2′)-Ia, ac(6′)-II*, Sulphonamide: *sul1*, Tetracycline: *tet(A)*

All known colistin mutations are confirmed (Poirel et al., 2017). Strain types and genes conferring resistance to other antimicrobials were obtained through BacPipe. The MIC values were obtained using Phoenix and SensiTest methodologies. *All *E. coli* ST were detected using the *E. coli* database 1 (Wirth et al., 2006).

### DNA Extraction and Sequencing

Genomic DNA was extracted from all isolates using a QIAamp^®^ UCP Pathogen mini kit (Qiagen, Düsseldorf, Germany) according to the manufacturer’s protocol. Briefly, from each isolate’s genomic DNA was purified, and later quantified using a Qubit dsDNA high sensitivity assay (Thermo Fisher, Waltham, United States). Libraries were prepared using Nextera XT and sequenced via (2 bp × 250 bp) v2 500 cycle, MiSeq (Illumina, Inc., United States). The raw sequences were subjected to a quality check and analysis using our in-house pipeline, BacPipe v.1.2.6 (Xavier et al., 2020). Briefly, the reads were quality assessed, trimmed, followed by *de novo* assembly (SPAdes), typing (MLST), genome annotation (Prokka), and resistance genes (ResFinder and CARD). The scaffolds were run through Plasmid Finder (Carattoli et al., 2014) to determine the incompatibility type of the plasmids. Insertion element (IS) families were analysed through IS Finder (Siguier, 2006). Genetic characterisation, chromosomal gene modifications and *mcr* gene based phylogenetic analysis were done using CLC Genomics workbench v9.5.3 (CLCbio, Denmark).

## Results

All isolates (*n* = 18) showed resistance to colistin with MICs > 4 μg/mL in both Phoenix and SensiTest results, except KPN-2 and EC-21 (Table 1). The clonal diversity among the isolates was determined; the *K. pneumoniae* isolates had nine different strain types (STs) (ST11, ST383, ST792, ST25, ST323, ST231, ST147, ST716, and ST2096, the *E*. *coli* isolates had two unique types (ST19, ST53), and one novel ST (Wirth et al., 2006), and *P. aeruginosa* had two types (ST500, ST357) (Table 1).

**FIGURE 1:**
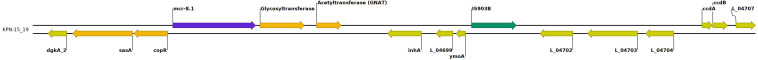
Genetic organisation of pKPN-15 and pKPN-19 harbouring *mcr-8.1* gene.

Two *K. pneumoniae* isolates (KPN-15 and KPN-19) harboured *mcr-8.1* (Figure 1), and one *E. coli* (EC-12) carried *mcr-1.1*. For the *mcr-8.1* harbouring strains, KPN-15 was found to have a MIC of > 16 μg/mL and KPN-19 a value of 16 μg/mL when evaluated using SensiTest. All other *K. pneumoniae* isolates had MIC values of 16 or > 16 μg/mL (Table 1). EC-12 harboured *mcr-1.1* and was observed to have a MIC value by SensiTest of 4 μg/mL.

The contigs containing the *mcr* genes were analysed using BLAST to determine their origin and genetic context (Figure 1). Contig from isolate KPN-15 gave the top BLAST search hit to plasmid *pLH94-8* (CP035204) with an 81% coverage and 99.7% sequence identity. Similarly, contig from isolate KPN-19, a top BLAST search hit was plasmid *pLH94-8*, with a 77% query coverage and 99.3% nucleotide identity. Isolates KPN-15 and KPN-19 harboured plasmid types (*IncFII(Yp)* and *IncL)* and *IncFII(K)*, respectively. *E. coli* isolate EC-12 underwent the same analysis procedure as the *K. pneumoniae* strains. Isolate EC-12 contained *mcr-1.1* with the top BLAST search hit being *E. coli p*1540-1 (CP019052) with 100% query coverage and 99.9% sequence identity. PlasmidFinder revealed the *mcr-1.1* was harboured on plasmid type *IncI2*. KPN-15 and KPN-19 containing *mcr-8.1* as well as the reference *pLH94-*8 were also examined for additional resistance genes to other antimicrobial agents and genetic similarity. The contigs containing the *mcr-8.1* gene, harbours no other resistance genes were identified for either KPN-15 or KPN-19. However, it was found that all isolates harbours other resistance genes such as beta-lactams, aminoglycoside, macrolides and tetracycline (Table 1).

While only three isolates (KPN-15, KPN-19, and EC-12) carried *mcr* genes, other genetic mutations that have been previously associated with colistin resistance were observed and are reported in Table 1. In particular, mutations in the negative PhoPQ regulator *mgrB*, as well as in *phoP*, were the main modifications in study isolates linked to colistin resistance. *K. pneumoniae* isolates (KPN-9, KPN-10, KPN-14, and KPN-15) had modifications in the *mgrB* gene. Three of these isolates (KPN-9, KPN-10, and KPN-15) had MIC values of > 16 μg/mL while KPN-14 had a value of 16 μg/mL. KPN-9 and KPN-10 had the gene disruption at position 74th nucleotide, while KPN-15 had it at position 118th nucleotide. For KPN-9 it was determined that ISKpn-26 mediated the disruption. No insertion element could be found for KPN-10, and the change appeared to be due to a frameshift mutation. For KPN-15, while the disruption was mediated by *IS1* family elements, the specific element could not be determined. Isolate KPN-20 appeared to be lacking the *mgrB* gene entirely, with only 10 bp remnants corresponding to the gene being found. While there were multiple mutations observed in the PhoPQ and PmrAB two-component systems in our isolates, only the *phoP* L26Q mutation was linked to colistin resistance. This mutation was present in all the *K. pneumoniae* isolates (*n* = 13). The evolutionary relationship of *mcr-8.1* were compared with all other known *mcr* genes (1-10) and its variants. The *mcr-8.1* gene is closely related to *mcr-4* and *mcr-5* genes (Supplementary Figure S1).

## Discussion

### Clonal Diversity and Plasmid Types

The clonal diversity of the isolates showed that the majority belonged to known clones. In particular, the ST for *K. pneumoniae* mostly belonged to known epidemic clones (ST147, ST11, ST231, ST792, ST383). For the isolates that harboured *mcr* genes (KPN15, KPN-19, EC-12), the ST type was examined to see if it had previously been linked to other antimicrobial-resistant mechanisms. KPN-15 belonged to ST383; a well-characterised ST known to harbour genes conferring carbapenem resistance (Sabirova et al., 2016), while KPN-19 was ST716, a strain type that has been previously linked to an outbreak of New-Delhi metallo-beta-lactamase resistant *K. pneumoniae* (Heinrichs et al., 2019). EC-12 belonged to ST156, reported previously in *mcr-1.1* harbouring *E. coli* in Brazil, and a carbapenemase carrying isolate in China (Rossi et al., 2017).

The incompatibility types of the plasmids found in these isolates harbouring *mcr-8.1* or *mcr-1.1* were searched in the literature to look for previous reports of *mcr* carriage. The presence of *IncFII* plasmids in KPN-15 and KPN-19 potential source of origin harbouring *mcr-8.1* gene variants has been reported in previous studies (Wang et al., 2018, 2019; Hadjadj et al., 2019; Wang et al., 2019). Similarly, the presence of *IncI2* in EC-12 is unsurprising as it is the first *Inc* type described with *mcr-1.1* and has been widely reported since then (Zurfluh et al., 2017).

### *mcr* Genes

Studies have shown that *mcr-1.1* confers low to moderate level resistance (Cao et al., 2018), as seen in isolate EC-12 which had a MIC value of 4 μg/mL. Previous studies on *mcr-8.1* have reported MIC values of 16 μg/mL (Wang et al., 2018; Bonnin et al., 2020), however, for our study, we are unable to conclude if our isolates demonstrate higher than 16 μg/mL as the SensiTest method can only delineate colistin MICs up to 16 μg/mL. Nonetheless, all of the resistant *K. pneumoniae* strains, whether or not they carried *mcr-8.1*, had values of 16 μg/mL or > 16 μg/mL (Table 1). Additionally, mutations to the PhoPQ system, such as the L26Q *phoP* mutation seen in all *K. pneumoniae* strains, confer low to moderate level (8-64 μg/mL) polymyxin resistance (Miller et al., 2011).

A recent paper by Farzana et al. (2020) on isolates from Bangladesh describes the presence of *mcr-8.1* on plasmid *IncFIB(pQil)* and is associated with a fitness cost. The study proposed that *mcr-8.1* inserted at the same site as carbapenem resistance genes in *IncFIB(pQil)* (Farzana et al., 2020). This paper also noted that there was evidence suggesting that IS903B elements mediated the initial insertion of *mcr-8.1* into the IncFIB(pQil) plasmid as these were seen to bracket all antimicrobial resistance elements in the studied strains (Farzana et al., 2020). IS903B has also been observed in other previously conducted studies on the *mcr-8.1* carriage in *K. pneumoniae* (Hadjadj et al., 2019). Interestingly, isolates KPN-15 and KPN-19, harbour IS903B. This suggests that IS5 family elements may have some role in resistance gene transfer but is inconclusive regarding *mcr-8.1* transfer specifically. For both our isolates and those in the Farzana et al. (2020) paper, the sequences flanking the *mcr-8.1* gene were examined for similarities. It was found that the genetic organisation of the contigs containing the *mcr-8.1* gene for our strains as well as the Farzana strains were highly similar. KPN-15/19 and the three Farzana strains share the same genetic context in the immediate flanking regions of *mcr-8.1*.

In contrast, the Farzana strains have approximately the entire length of the *mcr-8.1* harbouring plasmid in common and KPN-15 and KNP-19 share approximately 31740 bp in the *mcr-8.1* containing contigs (data not shown). Due to the pattern of similarities in the genetic organisation, it is hypothesised that all of the strains share a common *mcr-8.1* ancestor but are not evidence of clonal spread between Qatar and Bangladesh. Some of the key genes observed flanking *mcr-8.1* in all the strains were *copR* and *sasA*. In previous studies the presence of *copR* was also observed on a *K. pneumoniae pK19* plasmid from pig faeces and a *K. pneumoniae pLH94-8 IncFII* plasmid from a human sample (Wang et al., 2018; Hadjadj et al., 2019).

A recent study described in conference proceedings documented the first known case of *mcr-8.1* of human origin in Saudi Arabia and concluded that the resistance phenotype was due to not only *mcr-8.1* but also the presence of *mcr-1.1* (Hala et al., 2020). This corresponds to our findings that *mcr-8.1* did not appear to be the sole source of colistin resistance and worked in collaboration with *mgrB* modifications. Due to our findings of *mcr-8.1* and *mcr-1.1* in human isolates in Qatar, the Saudi Arabia findings are of concern as they indicate a rise in prevalence of these resistance mechanisms in the region. Due to this, it is imperative that wider screening be implemented in order to improve the detection of *mcr-8.1* and other variants in the region.

### Chromosomal Modifications Linked to Colistin Resistance

In particular, mutations in the negative PhoPQ regulator *mgrB*, as well as in *phoP*, were the main modifications in our isolates linked to colistin resistance. Mutations, deletions, disruptions, etc. in the *mgrB* gene have been linked to high levels of colistin resistance, particularly in *K. pneumoniae*. While multiple mutations were observed in the PhoPQ and PmrAB two-component systems in our isolates, only the *phoP* L26Q mutation has been implicated in colistin resistance (Uz Zaman et al., 2018). Interestingly, the *phoP* L26Q mutation was also present in previously reported *mcr-8.1* harbouring *K. pneumoniae* (Wang et al., 2018; Bonnin et al., 2020; Nabti et al., 2020). This question that role of this mutation in conferring resistance.

In a 2014 study, it was reported that *mgrB* genes are thought to play a significant role in the resistance phenotype (Cannatelli et al., 2014). A 2018 study by Pitt el al noted that *mgrB* disruptions at position 74 were the most common (Pitt et al., 2018). This disruption is also seen in our isolates KPN-9 and KPN-10. In particular, the disruption in KPN-9 was mediated by *ISKpn26*; the same IS element previously identified as the most common driver of mutation in *mgrB* in *K. pneumoniae* (Pitt et al., 2018). For isolate KPN-15 the *mgrB* gene was split at nucleotide 118 due to an insertion element of the IS1 family. Due to the use of short-read sequencing, the specific type of IS1 element responsible for this disruption could not be determined. The lack of *mgrB* in isolate KPN-20 suggests the complete disruption of the gene.

## Conclusion

In this study, we show that different genetic factors play a role in colistin resistance among isolates of *K. pneumoniae* from various clinical samples in a hospital in Qatar. The primary mechanism of colistin resistance remains via chromosomal mutations. However, we have identified *mcr-8.1* in *K. pneumoniae* and *mcr-1.1* in *E. coli* that harbour both *mcr* genes and known chromosomal mutations, more specifically a single amino acid change in *phoP*, the net contribution of which needs to be studied further. This data is complementary to previous studies indicating the emergence of *mcr-8.1* in humans, both globally and in the Middle East. This suggests a more widespread distribution of the gene and the necessity for wider screening to detect its presence in clinical centres.

## Data Availability Statement

All datasets associated with this study are included in the article/Supplementary Material. The sequences of all 18 isolates described in this study submitted to NCBI GenBank under the BioProject: PRJNA609881.

## Ethics Statement

Ethical approval (MRC-01-17-198) was obtained from the Medical Research Centre (MRC), Hamad Medical Corporation (HMC), Doha, Qatar.

## Author Contributions

SM-K conceptualised the study. SM-K, BX, and NE designed the study. NE, EI, HA-M, HY, MA, CL, and AA carried out the experimental work. BK and BX performed the sequencing and analysis. BK, BX, NE, and SM-K drafted the manuscript. All authors reviewed the manuscript.

## Conflict of Interest

The authors declare that the research was conducted in the absence of any commercial or financial relationships that could be construed as a potential conflict of interest.

## Supplementary Material

FIGURE S1 Evolutionary relationship of *mcr* genes: the complete protein sequences of *mcr* variants from the GenBank were aligned (clustalx) and used for building a maximum likelihood tree. Branch support value, in percent, indicates Bayesian posterior probability, followed by maximum likelihood bootstrap value (>70). iTOL (https://itol.embl.de/) was used to visualise the tree.

TABLE S1 Strain and clinical characteristics of the isolates described in this study.
