# Supplementary figures and images for: Identification of mcr-8 in Clinical Isolates From Qatar and Evaluation of Their Antimicrobial Profiles
Source: Front Microbiol. 2020 Aug 24;11:1954. doi: 10.3389/fmicb.2020.01954 (PMC7476323; doi:10.3389/fmicb.2020.01954)

Tree scale: 0.1

| bootstrap |      |
|-----------|------|
| ☆         | 70   |
| ☆         | 77.5 |
| ☆         | 85   |
| ☆         | 92.5 |
| ☆         | 100  |

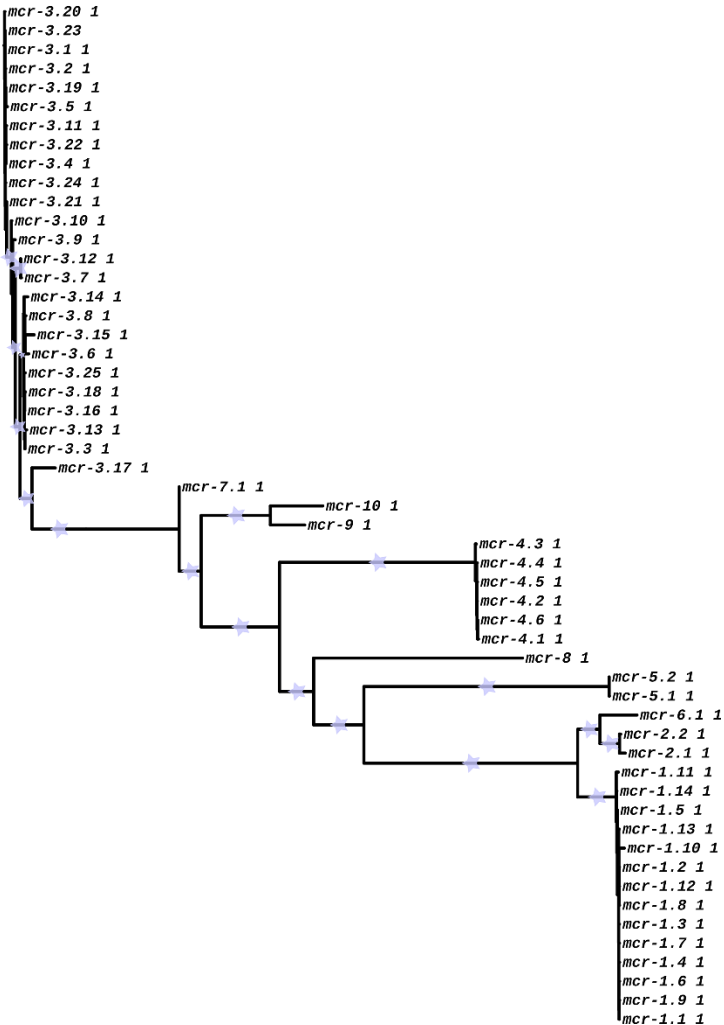

Supplement: FIGURE S1 — Evolutionary relationship of mcr genes: the complete protein sequences of mcr variants from the GenBank were aligned (clustalx) and used for building a maximum likelihood tree. Branch support value, in percent, indicates Bayesian posterior probability, followed by maximum likelihood bootstrap value (>70). iTOL (https://itol.embl.de/) was used to visualise the tree. [file Image_1.pdf]
