# Supplementary material for: Identification of mcr-8 in Clinical Isolates From Qatar and Evaluation of Their Antimicrobial Profiles
Source: Front Microbiol. 2020 Aug 24;11:1954. doi: 10.3389/fmicb.2020.01954 (PMC7476323; doi:10.3389/fmicb.2020.01954)
Supplement: TABLE S1 — Strain and clinical characteristics of the isolates described in this study. [file Table_1.DOCX]

| **Patient ID** | **Ethnicity** | **Age** | **Gender** | **Specimen type** | ***mcr* genes** |
| --- | --- | --- | --- | --- | --- |
| KPN-2 | Qatari | 34 | F | Wound | NA |
| KPN-3 | Qatari | 70 | M | Urine (Midstream) | NA |
| KPN-4 | Qatari | 76 | F | Urine (Bladder) | NA |
| KPN-5 | Palestine | 34 | M | Wound | NA |
| KPN-9 | Qatari | 69 | M | Urine (Catheter) | NA |
| KPN-10 | Qatari | 63 | M | Tracheal Aspirate | NA |
| PA-11 | Nigeria | 29 | M | Sputum | NA |
| EC-12 | Qatari | 61 | M | Wound | *Mcr-1.1* |
| PA-13 | Egypt | 47 | M | Tissue | NA |
| KPN-14 | Qatari | 22 | F | Tissue | NA |
| KPN-15 | Qatari | 70 | F | Urine (Catheter) | *Mcr-8.1* |
| KPN-16 | Qatari | 69 | M | Urine (Catheter) | NA |
| KPN-17 | Iraqi | 51 | F | Respiratory (endotracheal tube) | NA |
| KPN-18 | Pakistan | 57 | F | Wound | NA |
| KPN-19 | Indian | 34 | M | Bronchial Wash | *Mcr-8.1* |
| KPN-20 | Indian | 60 | F | Central line | NA |
| EC-21 | Qatari | 59 | F | Urine (Midstream) | NA |
| EC-22 | Qatari | 75 | F | Urine (Bladder) | NA |

**Supplementary Table 1**: Strain and clinical characteristics of the isolates described in this study
